# Phenotypic Plasticity of Southern Ocean Diatoms: Key to Success in the Sea Ice Habitat?

**DOI:** 10.1371/journal.pone.0081185

**Published:** 2013-11-21

**Authors:** Olivia Sackett, Katherina Petrou, Brian Reedy, Adrian De Grazia, Ross Hill, Martina Doblin, John Beardall, Peter Ralph, Philip Heraud

**Affiliations:** 1 School of the Environment and the Plant Functional Biology and Climate Change Cluster, University of Technology, Sydney, New South Wales, Australia; 2 School of Biological Sciences, Monash University, Victoria, Australia; 3 School of Chemistry and Forensic Science, University of Technology, Sydney, New South Wales, Australia; 4 Centre for Marine Bio-Innovation and Sydney Institute of Marine Science, School of Biological, Earth and Environmental Sciences, The University of New South Wales, New South Wales, Australia; 5 Centre for Biospectroscopy and School of Biological Sciences, Monash University, Victoria, Australia; University of Connecticut, United States of America

## Abstract

Diatoms are the primary source of nutrition and energy for the Southern Ocean ecosystem. Microalgae, including diatoms, synthesise biological macromolecules such as lipids, proteins and carbohydrates for growth, reproduction and acclimation to prevailing environmental conditions. Here we show that three key species of Southern Ocean diatom (*Fragilariopsis cylindrus*, *Chaetoceros simplex* and *Pseudo-nitzschia subcurvata*) exhibited phenotypic plasticity in response to salinity and temperature regimes experienced during the seasonal formation and decay of sea ice. The degree of phenotypic plasticity, in terms of changes in macromolecular composition, was highly species-specific and consistent with each species’ known distribution and abundance throughout sea ice, meltwater and pelagic habitats, suggesting that phenotypic plasticity may have been selected for by the extreme variability of the polar marine environment. We argue that changes in diatom macromolecular composition and shifts in species dominance in response to a changing climate have the potential to alter nutrient and energy fluxes throughout the Southern Ocean ecosystem.

## Introduction

Macromolecules, including proteins, lipids and carbohydrates are the building blocks of life. Microalgae are the primary source of macromolecules in marine ecosystems through their photosynthetic assimilation of dissolved inorganic carbon into organic carbon biomass that is then consumed by higher trophic levels. In the Antarctic, greater than 50% of this production is contributed by diatoms, which dominate the microalgal assemblage, ultimately providing food for krill, fish, whales, penguins, and seabirds [1]. This capacity to synthesise macromolecules enables microalgae to acclimate to prevailing environmental conditions. For example, lipids are synthesised to sustain membrane structure and function and for energy storage, while proteins and carbohydrates have wide-ranging uses including maintenance of cell walls, membrane structure and function, mucus production, and osmoregulation [2–4]. Such responses to environmental conditions have trophic implications because microalgal macromolecular composition affects herbivore assimilation efficiencies and reproductive success [5].

**Figure 1:**
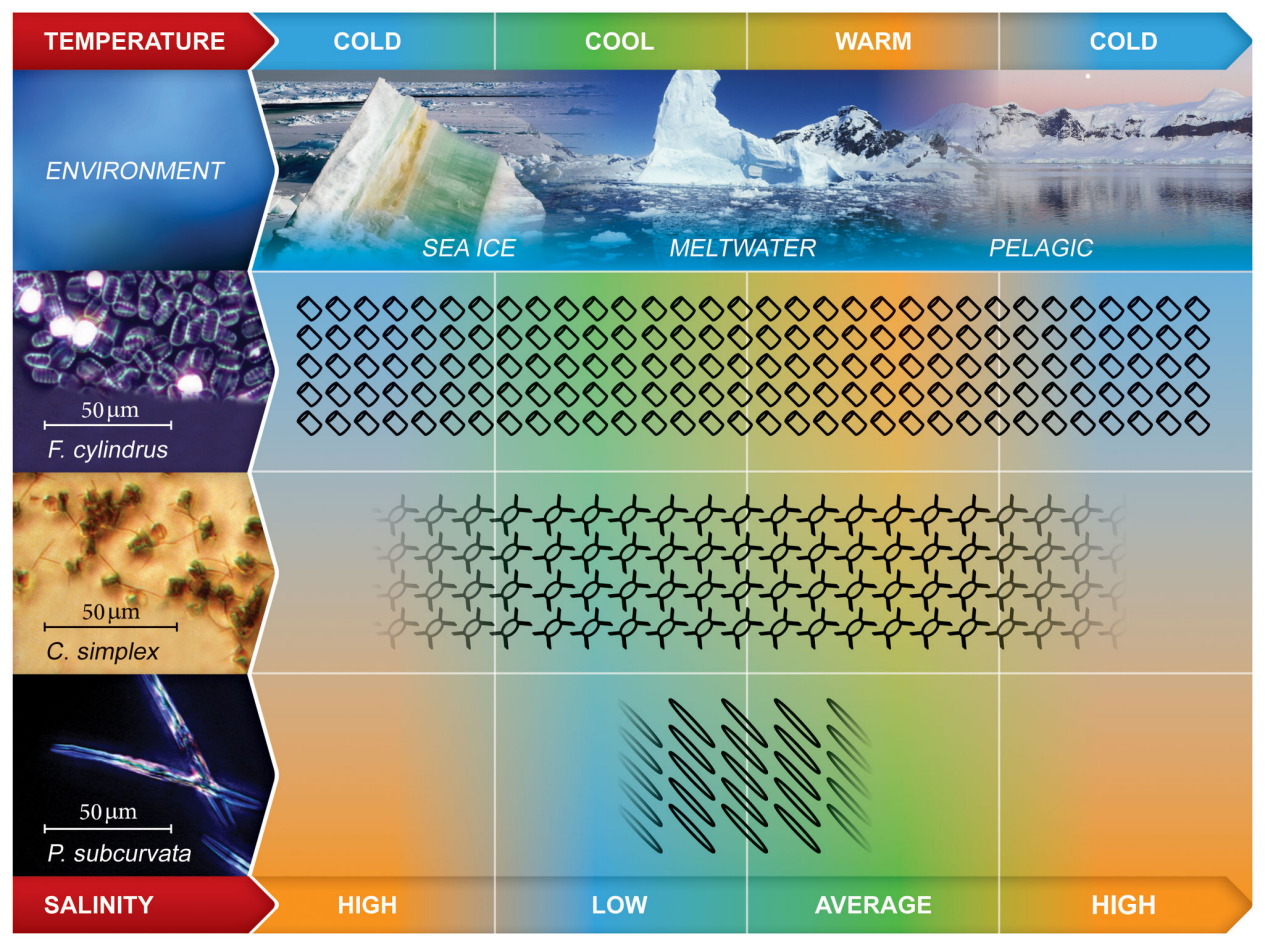
Major Southern Ocean habitats and related distribution of diatom species. The seasonal cycle of sea ice formation and decay produces three major habitats: sea ice, meltwater and pelagic. Sea ice brine channels contain cold, highly saline water within which extremophile microalgae, such as *F. cylindrus*, are able to flourish (visible as brown coloured regions within the sea ice). The melting sea ice produces a stable layer of cool, low salinity water to which species such as *P. subcurvata* have adapted. Once the stratified meltwater layer has broken down, deeper mixing returns and the pelagic habitat prevails. *C. simplex* is most successful in the pelagic and meltwater habitats, but also manages to survive the winter months within the sea ice. *F. cylindrus* is abundant in all three habitats but frequently dominates the sea ice microalgal community.

The Southern Ocean is characterised by the seasonal formation and decay of sea ice, which produces a range of environmental conditions from deeply mixed waters in summer to solid ice sheets in winter [6,7]. Consequently, diatoms experience rapid fluctuations in physical and chemical conditions associated with the transition from sea ice to meltwater and pelagic habitats (Figure 1) [8]. This highly variable habitat has driven the evolutionary adaptation of extremophile diatoms, which are capable of growing and photosynthesising under conditions that lie at the ends of temperature, pH and salinity tolerance scales [8,9].

Phenotypic plasticity refers to an organisms’ ability to change their chemistry, physiology, development, morphology or behaviour, to maximise fitness in variable environments [10]. Photophysiological plasticity in Southern Ocean diatoms in response to short term changes in salinity and temperature has previously been reported by Petrou at al. [8,11]. This study builds on the work conducted by Petrou et al. by investigating whether such environmental changes are also associated with plasticity in macromolecular composition.

Lipid accumulation has frequently been reported in sea ice microalgal communities from both the Arctic and the Southern Ocean and is thought to provide an important source of nutrition and energy to higher trophic levels [12,13]. Although phytoplankton blooms in the pelagic habitat usually contribute a greater percentage of annual primary production, sea ice microalgal communities are thought to provide a vital injection of lipid-rich biomass during the winter-months, when production by phytoplankton is limited by low light conditions under the sea ice [5,6]. The average caloric value of lipid biomass in microalgae is reportedly 1.98 and 2.26 times higher than that of protein and carbohydrate biomass, respectively [14]. Therefore factors that affect the production of these compounds can influence the overall caloric value of the cell [15]. Here we show that the magnitude of change in cellular lipid content is highly species-specific, demonstrating that shifts in community composition have the potential to alter the supply of energy to higher trophic levels [5,16]. Understanding such variability in macromolecular composition provides important information about acclimation strategies of microalgae. It is also essential if we are to understand how the nutritional value of the microalgal community may respond to environmental change, which has implications for the productivity of the entire Antarctic ecosystem.

Despite its importance, we have little understanding of macromolecular composition of individual microalgal species within natural assemblages. This is primarily due to technical limitations of traditional bulk analysis techniques, which do not have the capacity to target individual species within mixed communities. Fourier Transform Infrared (FTIR) microspectroscopy is a vibrational spectroscopic technique becoming increasingly popular amongst microbial ecologists [17,18]. The technique measures concentrations of macromolecules and can be used for the classification of samples based on their infrared spectral “fingerprint” [19]. When coupled with a synchrotron light source, infrared microspectroscopy achieves high sensitivity and spatial resolution (~3 μm for lipids), enabling the analysis of individual microalgal cells [20–22]. FTIR microspectroscopy is therefore an excellent candidate for collecting species-specific data from natural populations, because it allows the targeting of individual species within a mixed sample.

To improve our understanding of macromolecular plasticity and associated changes in nutritional value in response to salinity and temperature regimes characteristic of sea ice, meltwater and pelagic habitats, FTIR microspectroscopy was used to measure macromolecular concentrations in three Antarctic diatoms: *Fragilariopsis cylindrus*, a dominant species in the sea ice and ice-edge habitats of both polar regions [23]; *Chaetoceros simplex*, which is found in great abundance in pelagic and meltwater habitats around Antarctica and belongs to the most abundant algal genus on the planet [24]; and *Pseudo-nitszchia subcurvata*, typically found in meltwater habitats of coastal Antarctic waters [6,25]. This study tests the hypotheses: i. that species well adapted to life in the sea ice demonstrate high phenotypic plasticity in terms of changes in macromolecular composition in response to the highly variable polar marine environment, and ii. that the nutritional value of Antarctic diatoms is influenced by the fluctuating salinity and temperature conditions experienced during an annual cycle. For the first time, synchrotron FTIR microspectroscopy and multivariate data analysis and modelling are used to measure the macromolecular composition of individual Antarctic diatom cells and quantitatively assess the degree of phenotypic plasticity of cell populations in response to conditions characteristic of sea ice meltwater and pelagic habitats.

## Materials and Methods

### Culturing

*F. cylindrus* (Grunow) was collected from ice cores (66°S, 147°E) taken in November 2001. Cultures of the Antarctic diatom *C. simplex* were isolated from the coastal waters of Antarctica, Prydz Bay (CS 624, Australian National Algae Culture Collection, CSIRO, Hobart, Australia) and *P. subcurvata* (Hasle) Fryxell was collected from the subpolar South Atlantic Ocean. No permission was necessary for the collection of the algal samples from the Southern Ocean, however permits were obtained from the Australian Quarantine and Inspection Service (AQIS) for the import of samples into Australia. The taxonomic classification of all species was confirmed, based on analysis of frustule morphology by light microscopy. All species were maintained in approximate pelagic conditions at 4°C in natural Antarctic filtered seawater (0.2 µm, 0.28 nM Fe, 145.9 °S 54.0 °S, 72 m), collected during SAZ-Sense, Jan.- Feb. 2007, RV Aurora Australis. Culturing was conducted in specially designed 1 L glass culturing vessels using natural seawater (salinity 34) enriched with F/2 nutrients, with continuous air bubbling and maintained at +4°C (Guillard and Ryther 1962). Cultures received a 16:8 h light:dark cycle at 50 µmol photons m-2 s-1 (Grolux, GMT lighting, Northmead, Australia). Exponential growth phase (confirmed with regular in vivo auto-fluorescence measurements as a proxy for biomass [26]; Trilogy, Turner Designs Inc., Sunnyvale CA, USA) was maintained for the duration of the experiment by diluting (up to 90%) with fresh medium every 3-5 days. Cultures were concentrated by gentle vacuum filtering using 2 µm polycarbonate membrane filters (Millipore, MA, USA). Concentrated cultures were re-suspended in approximately 150 mL of medium in 250 mL culture flasks at three different salinities (31, 34 and 70 (± 0.5), approximating the characteristics of meltwater, pelagic and sea ice brine channels respectively) and stoppered with gauze (n=4). The salinity of the F/2 medium was adjusted either by the addition of MilliQ water or sodium chloride salt (Sigma, USA) and measured by refractometer. Flasks were then transferred to a temperature-controlled incubator and maintained at one of three temperatures (-1.8, +2 and +5°C (± 0.3°C), simulating characteristic of sea ice, meltwater and pelagic habitats) where the cells were given 72 h (well within the previously reported 7-day cold acclimation phase for *F. cylindrus*) to acclimate before samples were removed and analysed [27]. Pulse amplitude modulated (PAM) fluorometry was used to confirm the algae were extant and photosynthetically active prior to all other measurements being taken.

### FTIR Microspectroscopy

Approximately 15 mL of cell suspension was filtered through 1 µm polycarbonate filter membranes using a hand-operated vacuum filter tower. Cells collected on the filter were then resuspended in isotonic saline solution (NaCl (Sigma, USA) and MilliQ water) to wash the cells to remove F/2 medium which contains compounds that can absorb infrared radiation and possibly confound the FTIR measurements [18]. The saline solution was kept at the same temperature as the incubation temperature of the cells. This rinsing process was repeated three times for each replicate. Washed cells were deposited on Kevley MirrIR Low-e Microscope Slides (Kevley Technologies, Ohio, USA) using a Shandon Cytospin Centrifuge (Cytospin III, Thermo Fischer Scientific, Waltham, MA) and immediately stored in a vacuum desiccator at room temperature until analysis [18].

Spectral data were collected on the Infrared Microspectroscopy Beamline (2BM1B) at the Australian Synchrotron, Melbourne, Australia in August 2010 (time between experimental culturing and synchrotron spectroscopy of dried material was less than 1 year). Spectra were acquired over the measurement range 4000-800 cm^-1^ with a Vertex 80v FTIR spectrometer (Bruker Optics, Ettlingen, Germany) coupled with an IR microscope (Hyperion 2000, Bruker) with a Mercury Cadmium Telluride detector cooled with liquid nitrogen. The microscope was connected to a computer-controlled microscope stage and placed in a specially designed box which was purged with dehumidified air. The measurements were performed in the mapping mode, using a nominal aperture size of 5 µm × 5 µm with a spectral resolution of 8 cm^-1^, with 64 scans co-added. Adjusting the aperture for each species provided spectra that were representative unambiguously of individual cells (not clumps of cells) for *C. simplex* and *P. subcurvata*. However, for *F. cylindrus* spectra were acquired from groups of 5-10 cells due to a lower signal to noise ratio compared with the other species. The number of co-added scans was chosen as a good compromise between achieving spectra with good signal to noise characteristics and the rapid acquisition of data. Each treatment had four replicates and measurements were taken from a minimum of 50 cells per replicate. Spectra were processed using Happ-Genzel appodization and 2 levels of zero-filling. Spectral acquisition and instrument control was performed using Opus 6.5 software (Bruker).

FTIR spectral data was exported from the OPUS 6.5 for multivariate analysis using The Unscrambler X v 10.2 (Camo Inc., Oslo, Norway). An initial quality control procedure was performed over the range 3000-950 cm^-1^ where spectra with maximum absorbance greater than 0.85, which resulted from spectral acquisition of regions of the sample where cells were clumped, were rejected. Spectra were then pre-processed taking the second derivative using the Savitzky-Golay algorithm with 9 smoothing points, and normalization using Extended Multiplicative Signal Correction (EMSC).

### Multivariate modeling

Partial Least Squares Discrimination Analysis (PLS-DA) modelling was used to classify samples by experimental treatment conditions and species based on their spectra using the Non-linear Iterative Partial Least Squares (NIPALS) algorithm on mean-centred data. Discriminant analysis based on the PLS approach is useful for dealing with complicated data sets where ordinary regression is difficult or impossible to apply [28]. The NIPALS algorithm allows for linearization of models which are non-linear in the parameters [28]. Since it was first published in 1966, the PLS approach has become a standard tool in chemometrics and a superior method for discriminant analysis over Principal Component Analysis [29].

For PLS-DA, infrared spectra from 200-300 individual cells was used to generate a PLS-DA model which was then validated using a test set of 200-300 individual cell spectra which were not used to generate the PLSDA models. The PLSDA training data matrix comprised the spectra (X-variables) and three Y variables with integer values of 0 or +1 coding for the three modelled spectral classes (sea ice, melt water or pelagic conditions, respectively; or the three different species)^49,50^. PLSDA used the spectral ranges containing bands of biological origin between 3050-2800 and 1780-1000 cm^-1^. Spectra with a predicted Y-value of ≥0.5 were accepted as being from cells of the relevant species or treatment; spectra with predicted Y-values ≤0.5 were rejected. To assess the validity (accuracy) of each model, s*ensitivity* and *specificity* statistics were calculated using the equations True Positives/(True Positives+False Negatives) and True Negatives/(False Positives+True Negatives) respectively [30].

Partial Least Squares Regression (PLSR) analysis was used to compare variations in infrared spectra between the ranges 3050-2800 and 1780-1000 cm^-1^ from 200-300 individual cells (X-variables) in response to both temperature and salinity conditions (Y-variables). PLSR analyses were conducted on mean-centred data using the NIPALS algorithm and validated using an independent test set of 200-300 samples which were not used to generate the PLSR models.

Relative changes in the concentration of lipids, carbohydrates, amino acids and phosphorylated molecules were estimated based on proportionality between absorbance and analyte concentration according to the Beer-Lambert Law, previously demonstrated with diatoms and other types of microalgal cells [31–33]. Protein concentration was determined using a combination of mass spectrometry and FTIR spectroscopy to build a predictive PLSR model. Firstly, protein concentration was determined in a subset of *C. simplex* samples using mass spectrometry based on the previously reported nitrogen to protein conversion factor for microalgae of 4.78 [34]. Because protein represents a small fraction of the dry mass of diatoms relative to the mass of the silicate frustule, protein was reported as the percentage of total carbon contributed by protein. Protein from *C. simplex* was previously reported to consist of 52% carbon [35]. The calculation of the percentage total carbon contributed by protein was as follows:

$$\begin{array}{c} C_{protein(g)}=\frac{N_{\left(g\right)}\times 4.78}{0.52}\\ C_{protein(\%C_{total})}=\frac{C_{protein(g)}}{C_{total(g)}}\times 100 \end{array}$$

Where C_protein_ is the mass of C from protein, C_total_ is the total mass of carbon and N is the mass of nitrogen per unit dry weight.

Protein measurements were then used as a training set to build a PLSR model based on 499 cell spectra. A further 295 cell spectra were used to validate the model. Experimental replicates that were used in the training data set were not used in the validation data set. This PLSR model was subsequently used to predict the protein carbon (as percent of total carbon) for the remainder of the samples.

### Statistical analyses

Statistical analyses were conducted using SigmaPlot for Windows version 11.0 (Systat Software, Inc). If the data did not meet the assumption of homoscedasticity then a Kruskal Wallis on ranked data was used to replace the one-way ANOVA. In the case of a two-way ANOVA where data did not meet the assumption of homoscedasticity or normality, a non-parametric Scheirer-Ray-Hare test was used on ranked data instead. The Scheirer-Ray-Hare test was performed on Predictive Analytical Software (Version 18, SPSS Inc, Chicago, IL, USA) with additional calculations by hand and a Chi-squared table for determining the P-value. Given the reduced power of the S-R-H test, a more conservative significance level was set at α = 0.01 for these data. For all other analyses, significance level was set to α = 0.05.

## Results

Microalgal cells from each of the three species (*F. cylindrus, C. simplex and P. subcurvata*) were exposed to three levels of salinity and temperature in order to simulate sea ice (70, -1.8°C), meltwater (30, 2°C) and pelagic (34, 5°C) habitats. Changes in macromolecular composition were then measured using FTIR spectroscopy. Data are freely available from the authors upon request.

### Degree of Phenotypic Plasticity Varies between Diatom Species

The degree of plasticity was determined by comparing the accuracy of Partial Least Squares Discriminant Analysis (PLSDA) models used to classify “unknown” samples by treatment conditions. Samples which had a macromolecular composition more highly differentiated would be classified with a higher degree of accuracy than those which were more similar. *F. cylindrus* and *C. simplex* displayed a higher degree of macromolecular plasticity (sensitivity of classification 85.7-97.6%; Table 1) in response to changes in salinity and temperature conditions, relative to *P. subcurvata* (sensitivity of classification 42.8-77.9%). This was indicative of a greater magnitude of change in macromolecular composition in *F. cylindrus* and *C. simplex* than *P. subcurvata*, as outlined below.

**Table 1:** PLSDA Classification by Treatment Summary Statistics.

Species	*n*	Treatment	R^2^	Sensitivity (%)	Specificity (%)
*F. cylindrus*	201	Meltwater	0.754	94.9	99.4
		Pelagic	0.715	95.4	99.3
		Sea ice	0.591	97.6	91.2
*C. simplex*	295	Meltwater	0.560	96.7	89.5
		Pelagic	0.651	96.5	92.3
		Sea ice	0.782	85.7	100.0
*P. subcurvata*	294	Meltwater	0.268	77.9	86.4
		Pelagic	0.249	42.8	93.1
		Sea ice	0.532	74.0	97.2

### Source of plasticity: changes in macromolecular composition

**Figure 2:**
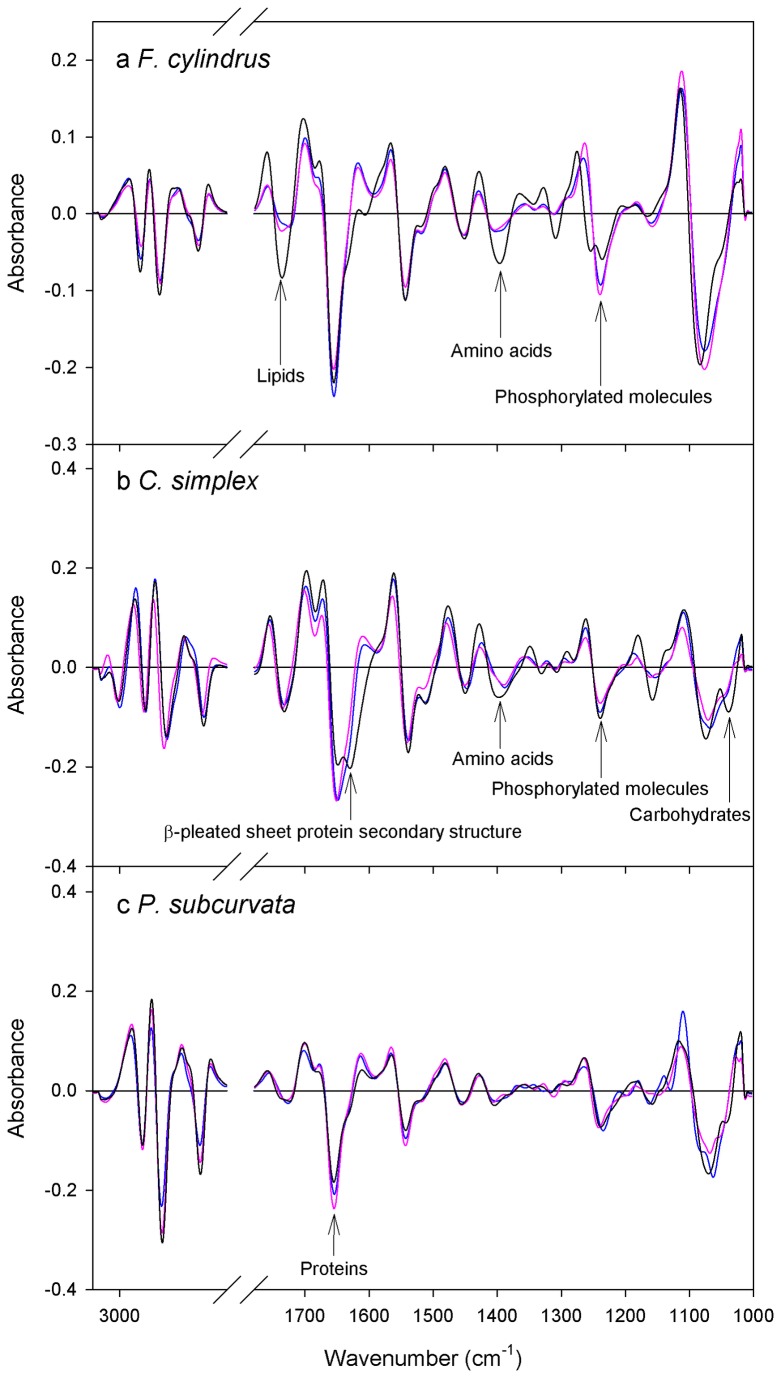
Average second derivative spectra. The average second derivative spectra of *F. cylindrus* (a), *C. simplex* (b) and *P. subcurvata* (c) from meltwater (blue), pelagic (pink) and sea ice (black) treated cells. Labels indicate the macromolecular pool represented by peaks in each spectrum. Standard error ranged from 9.16 × 10^-7^ to 1.76 × 10^-4^ absorbance units for the pooled dataset.

Macromolecular composition was different between treatments for all three species, with variations in maximum absorbance for bands corresponding to biological macromolecules (Figure 2; for band assignments see Table S1). *F. cylindrus* and *C. simplex* showed broad-scale changes in macromolecular composition including variation in levels of lipids (~1730 cm^-1^), proteins (~1650 cm^-1^), amino acids (~1400 cm^-1^) and phosphorylated molecules (~1250 cm^-1^), with a particularly strong increase in amino acid content evident for both species in the sea ice treatment (Figure 2a & b). Additionally, the appearance of a second peak in the Amide I region at ~1630 cm^-1^ indicated that *C. simplex* underwent distinctive changes in protein composition (Figure 2b). In contrast, changes in macromolecular composition in *P. subcurvata* were mainly restricted to proteins, which were at highest concentration in the pelagic and lowest in the sea ice treatment (Figure 2c).

**Figure 3:**
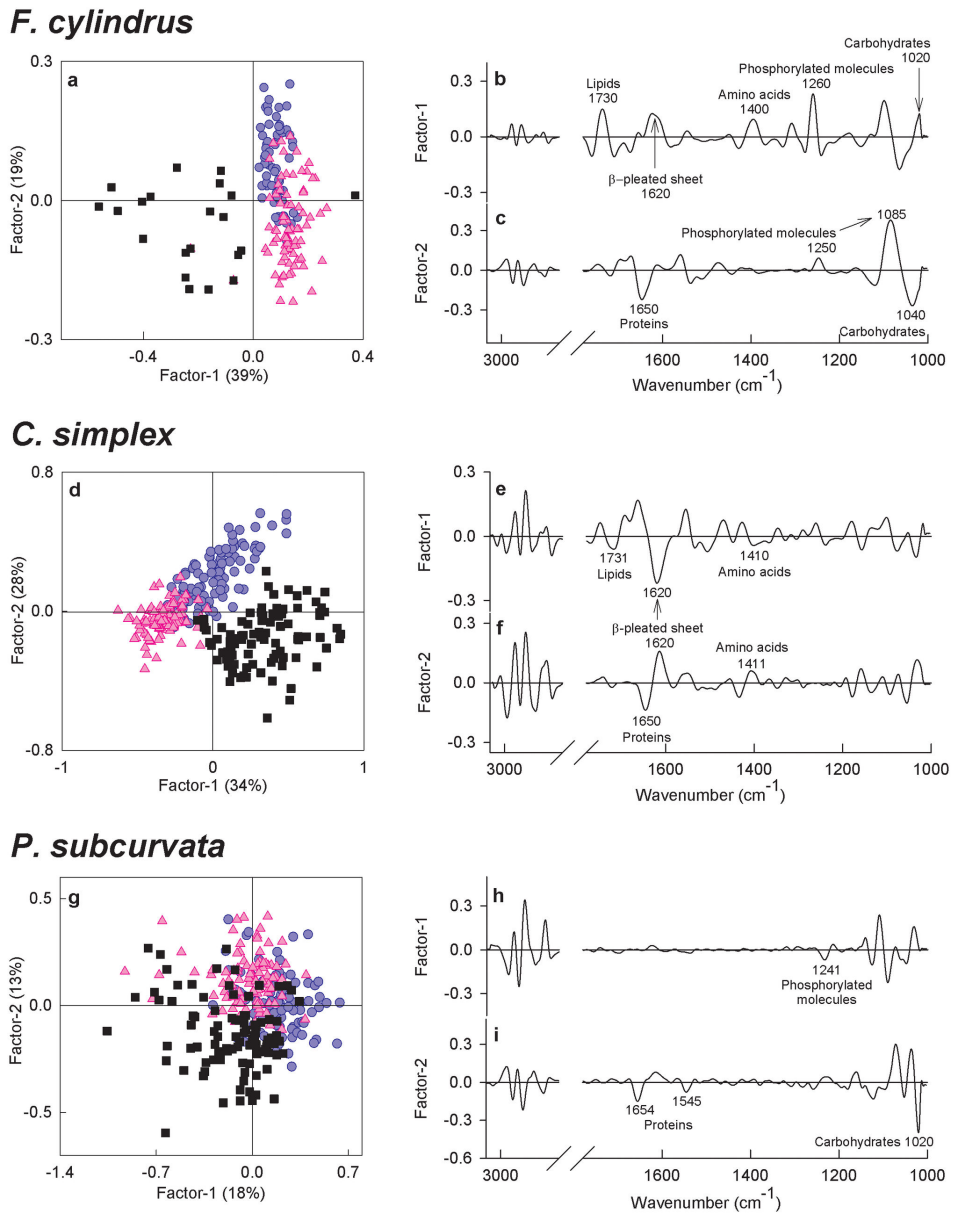
Partial Least Squares Discriminant Analysis (PLSDA). PLSDA modelling was used to classify samples by meltwater (●), pelagic (▲) and sea ice (■) treatment conditions based on their infrared spectra. Scores plots for *F. cylindrus* and *C. simplex* (a & d) clearly show three clusters of cell spectra, indicating that the macromolecular composition was distinctly different between treatments, whereas *P. subcurvata* showed minimal plasticity (g). Because the data were second derivative transformed, a positive peak in the loadings plot indicates a decrease in absorbance for points that have positive scores in the PLSDA scores plots.

PLSDA scores plots provided a visualisation of clustering patterns in the data set, intrapopulation variability (where each point represented an entire spectrum from an individual cell, or in the case of *F. cylindrus* a cluster of a few cells) and revealed more details about patterns of variation in macromolecular composition than could be determined from the average spectra (see Figure 3a, d & g). The PLSDA factors summarised variation in the data set, with the largest amount of variance being captured by Factor-1 and each subsequent factor capturing a lesser amount of variation. The loadings plots associated with the clustering patterns observed in the scores plots indicated which peaks in the spectrum drove variability between samples (Figures 3b, 3c, 3e, 3f, 3h, & 3i). For *F. cylindrus*, the Factor-1 loadings plot explained 39% of variation between cells and confirmed that levels of lipids (1730 cm^-1^), amino acids (1400 cm^-1^) and phosphorylated molecules (1260 cm^-1^) were higher in cells from the sea ice treatment compared to the meltwater or pelagic treatments, as shown in the average second derivative spectra (Figure 3a & b). Additionally, a prominent and broad band at 1620 cm^-1^ indicted that changes in protein composition occurred between treatments. The Factor-2 loadings plot explained 19% of variation between cells and indicated that cells from the meltwater treatment had increased protein (1660-1540 cm^-1^) and carbohydrate (1040cm^-1^) content and decreased levels of phosphorylated molecules (1250cm^-1^) compared to the pelagic treated cells (Figure 3a & c). Loadings plots for *C. simplex* showed fewer prominent peaks than for *F. cylindrus*, indicating that differences between cells were related to variations in fewer macromolecular pools. Factor-1 explained 34% of the variation between cells and was consistent with the average second derivative spectra (Figure 2b), whereby cells from the sea ice treatment showed higher levels of lipids (1731 cm^-1^) and distinct changes in protein composition (1668-1558 cm^-1^; Figure 3e). Factor-2, which explained 28% of the variation between cells, showed that protein compositional changes occurred between meltwater and pelagic treatments (Figure 3f). In the PLSDA scores plot for *P. subcurvata*, clustering of points by treatment was less distinct than for the other two species, indicating that much of the variability between cells was related to intra-population variation (Figure 3g). The loadings plots for *P. subcurvata* were characterised by fewer prominent peaks than either of the other two species, confirming that changes were limited to fewer macromolecular pools (Figure 3h & i). In combination, Factors-1 and 2 explained only 31% of variation between cells with moderate peaks at 1241, 1654, 1545 and 1020 cm^-1^ indicative of small variations in phosphorylated molecules, proteins and carbohydrates relative to the other two species. 

### Predictions of environmental history of cells

To further quantify the degree to which macromolecular composition varied with treatment for each species, Partial Least Squares Regression (PLSR) modelling was used to predict salinity and temperature levels based on cell spectra. A higher degree of accuracy indicated a more dissimilar macromolecular composition between treatments and hence a higher degree of plasticity for that species. Salinity and temperature could be predicted with accuracies of ± 12.1 and ± 2.6 °C, respectively, for *F. cylindrus* and ± 13.2 and ± 2.7 °C for *C. simplex* (Table 2). In contrast, models for *P. subcurvata* were almost 50% less accurate (± 25.7 and ± 4.2 °C for salinity and temperature, respectively), suggesting a lower degree of plasticity in macromolecular composition in response to environmental changes in this organism.

**Table 2:** PLSR Prediction Summary Statistics.

Variable	n	Species	R^2^	Accuracy (±)
Salinity	203	*F. cylindrus*	0.811	12.1
	295	*C. simplex*	0.776	13.2
	295	*P. subcurvata*	0.604	25.7
Temperature (°C)	201	*F. cylindrus*	0.756	2.6
	295	*C. simplex*	0.753	2.7
	295	*P. subcurvata*	0.556	4.2

### Change in concentration of macromolecules

**Figure 4:**
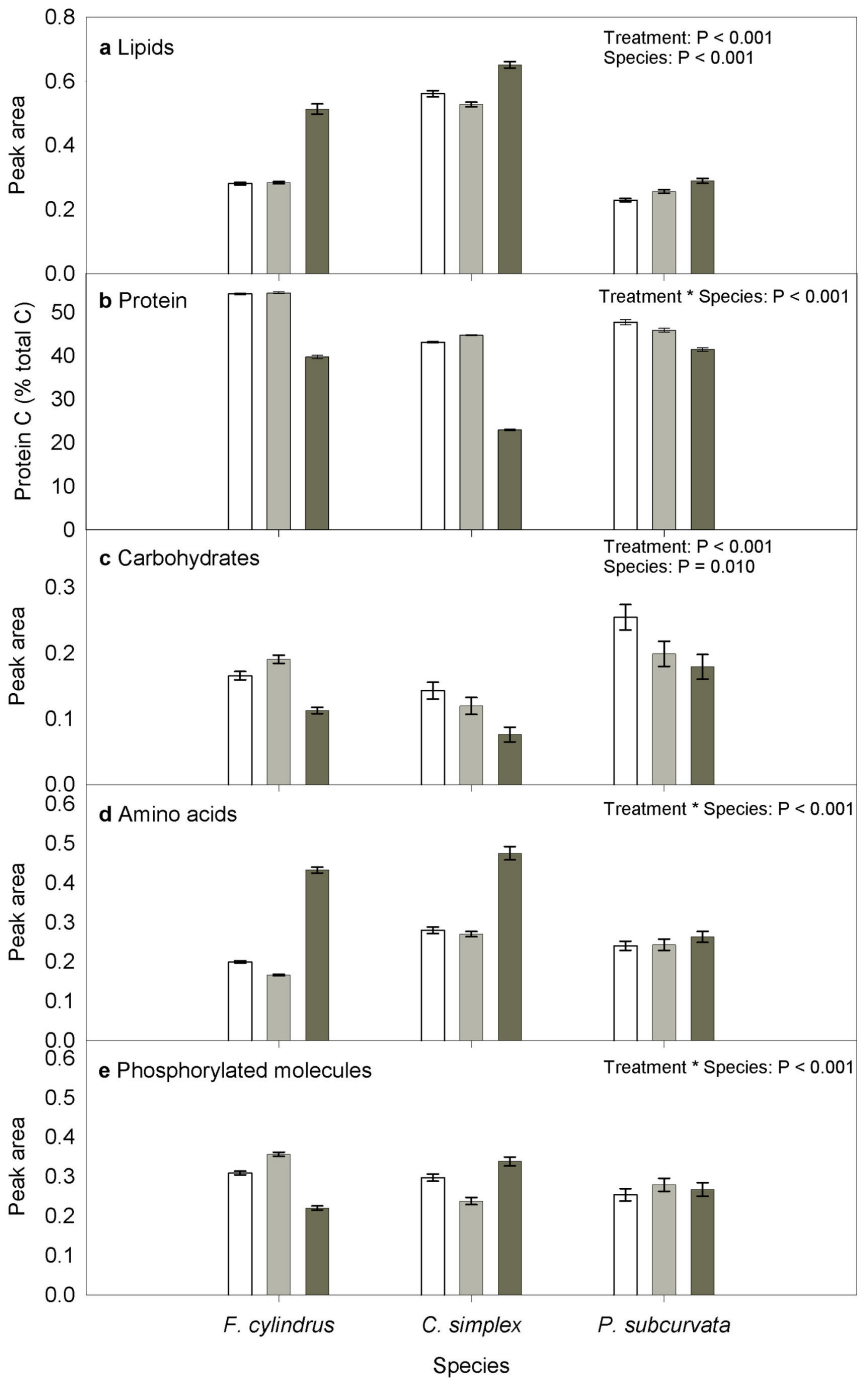
Cellular concentrations of macromolecules. Concentration is proportional to the area under the peak in the infrared spectrum corresponding to each macromolecule, according to the Beer-Lambert Law proportionality between concentration and absorbance. For all parameters excluding protein, bars show the mean peak area. Protein is expressed as the percentage of total carbon contributed by protein, determined by mass spectrometry for a subset of *C. simplex* samples (pelagic and sea ice treated samples). This mass spectrometry data was used as a Partial Least Squares Regression (PLSR) calibration data set (499 spectra) to predict protein content in the remaining samples based on their FTIR spectrum (1328 spectra). Key: meltwater (white), pelagic (light grey) and sea ice (dark grey) treatments. Error bars indicate the standard error. Results from Scheirer-Ray-Hare two-way non-parametric statistical tests are shown in the top right corner of each plot.

Lipid content increased under sea ice conditions in all three species (SRH test, P<0.001). *F. cylindrus* showed the greatest magnitude of change, with lipid content nearly doubling in the sea ice treatment compared to the meltwater and pelagic treatments (Figure 4a). *C. simplex* had the highest lipid content of the three species, regardless of treatment (SRH test, P<0.001). Protein content varied between treatments with the pattern of variation being different for each species (Figure 4b).Protein content was lowest in the sea ice treatment for all three species (two-way ANOVA, P<0.001).. Sea ice conditions resulted in the lowest carbohydrate content for all three species (SRH test, P=0.010). *P. subcurvata* had the highest and *C. simplex* the lowest carbohydrate content regardless of treatment (Figure 4c; SRH test, P<0.001). Amino acid content was significantly higher under sea ice conditions for both *F. cylindrus* and *C. simplex* (Figure 4d; SRH test, P<0.001) whereas *P. subcurvata* showed minimal variation in amino acid content. *F. cylindrus* demonstrated the greatest magnitude of change in amino acid content with levels more than doubling in the sea ice treatment compared to the meltwater and pelagic treatments. Phosphorylated molecules varied with both species and treatment (Figure 4e; SRH test, P < 0.001). The lowest levels of phosphorylated molecules occurred in the sea ice treatment for *F. cylindrus* and the pelagic treatment for *C. simplex*, whereas *P. subcurvata* showed minimal variation between treatments.

## Discussion

This study compared the phenotypic plasticity of three ecologically important species of Southern Ocean diatoms in response to salinity and temperature regimes characteristic of the highly variable polar marine environment. Photophysiological plasticity in response to these conditions was previously reported in Petrou et al. [11]. This paper builds on Petrou et al. by showing that phenotypic plasticity was also manifest as changes in macromolecular composition. Responses were species-specific in terms of which macromolecular pools varied (proteins, lipids, carbohydrates, amino acids and/or phosphorylated molecules) and the degree of plasticity. *F. cylindrus* and *C. simplex*, two diatom species with widespread distribution in diverse habitats in the Southern Ocean, each displayed a high degree of phenotypic plasticity, in terms of macromolecular composition, in response to changing temperature and salinity. In contrast, *P. subcurvata*, a meltwater specialist [25], displayed a comparatively low degree of plasticity. Additionally, reduced photosynthetic efficiency in *P. subcurvata* under sea ice treatment conditions provided further evidence that this species is less well adapted to the sea ice environment [11]. Together, these results suggest that phenotypic plasticity as shown here by *F. cylindrus* and *C. simplex* is advantageous under the highly variable physicochemical conditions associated with the seasonal formation and decay of sea ice.

Microalgae vary macromolecular pools to satisfy physiological requirements. Lipids are used as storage products, for the repair and maintenance of membranes [3,12,36–39]. Macromolecules such as anti-freeze proteins, ice-binding proteins and ice-active substances are known to be critical for osmoregulation and cryoprotection (preventing injury to cells due to ice crystal formation) [4,23,40–42]. The elevated lipid levels displayed by *F. cylindrus* and *C. simplex* in response to the sea ice treatment are consistent with previous observations of lipid accumulation by diatoms in the sea ice. *P. subcurvata* did not display a similar increase in lipid concentration.

Protein concentration decreased in all three species in the sea ice treatment. In addition, *C. simplex* and *F. cylindrus* showed a distinct change in protein secondary structure (indicated by a shift in the Amide I peak maxima from 1650 to between 1630 and 1616 cm^-1^) attributed to a decrease in α-helix dominated proteins and an increase in proteins with β-pleated sheet. Antarctic microalgae have previously been reported to contain high amounts of Ribulose-1,5-bisphosphate carboxylase/oxygenase (RUBISCO), the enzyme which catalyses carboxylation and oxygenation of ribulose-1,5-bis- phosphate (RuBP) in the chloroplast [43]. The authors suggested that the increased production of RUBISCO in psychrophilic species, relative to mesophilic microalgae, compensates for the reduced activity of the enzyme at low temperatures. Given that RUBISCO is dominated by beta-pleated sheet structure [44] the observed shift in the Amide I peak maxima in *F. cylindrus* and *C. simplex* (the two psychrophilic diatoms) cell spectra in the sea ice treatment is consistent with an increased proportion of RUBISCO in the protein pool. Cell spectra from *P. subcurvata* did not indicate similar changes in protein secondary structure.

Both *F. cylindrus* and *C. simplex* showed elevated levels of free amino acids under sea ice treatment conditions, whereas *P. subcurvata* did not. Proline is known to be a key osmoregulator in *Fragilaropsis* *sp.* and sea ice diatoms in general [41]. Hence, the increase in amino acid content shown by *F. cylindrus and C. simplex* in study is consistent with previous research indicating that proline production is likely to be another important adaptive response to life in the sea ice habitat. Additionally, the catabolism of proline upon cessation of salt stress releases a great deal of energy which is thought to assist with the cells’ physiological recovery [45].

Phenotypic plasticity can have significant ecological and genetic costs, which can reduce a species' fitness in comparison to a species with a less plastic phenotype [10]. The costs relate to the maintenance of genetic material encoding particular traits. Just as environmental instability has selected for phenotypic plasticity in *F. cylindrus* and *C. simplex*, the stability of the meltwater habitat may have selected against phenotypic plasticity in *P. subcurvata*, resulting in a reduced capacity to vary macromolecular composition in response to environmental conditions, as shown in this study. For comparison, the extremophile alga *Chlamydomonas raudensis* UWO241 which was isolated from a permanently ice-covered lake has also demonstrated a high level of phenotypic plasticity[46]. Although conditions under the ice covered lake are considered relatively ‘stable’, the combination of high salinity, low temperature and low light levels result in an extreme environment and select for phenotypic plasticity. In contrast, the meltwater environment is characterised by relatively favourable light and salinity conditions, which promote the rapid growth of algal blooms. Recent research highlights that some species have the ability to sustain long-term acclimation to environmental change through phenotypic variation alone [47,48]. Therefore, species with higher levels of phenotypic plasticity, such as *F. cylindrus* and *C. simplex*, may have greater resilience to environmental fluctuations associated with global climate change than species such as *P. subcurvata* [46].

As the Antarctic region warms due to global climate change, polar inhabitants will experience warmer winters with thinning and retreat of sea ice cover [49]. Such environmental change will likely alter the species assemblage composition, as conditions become more or less favourable to individual species [50,51]. Indeed, ‘massive’ blooms of *Chaetoceros* and *Fragilariopsis* species have already been observed in the Arctic growing under ice between 0.5 and 1.8 m thick in the Chukchi Sea [52]. The authors concluded that the under ice bloom was facilitated by increased penetration of light through the ice, which was thinner than previous years, had a high surface melt pond fraction and transmitted four-fold more incident irradiance than adjacent, melt pond-free ice. The elemental stoichiometry of the microalgal community is known to reflect the average stoichiometry of individual species [50]. Similarly, the average macromolecular composition of the microalgal community is determined by the macromolecular composition of the constituent species, which has been shown here to be highly species-specific. Hence, shifts in the assemblage composition will likely alter the average macromolecular composition of the microalgal community with potential implications for the quality of food supplied to the Southern Ocean ecosystem. Indeed, recently observed shifts in microalgal and zooplankton community composition along the West Antarctic Peninsula, one of the most rapidly warming regions on earth, have coincided with changes in krill recruitment, abundance and availability to predators [53].

Taxon-specific studies of microalgal physiology and associated effects on the nutritional value of natural populations are rare, particularly from the Southern Ocean [21,54–56]. Such data is in great demand for the calibration and validation of plankton functional type models used to predict how microalgal assemblages will respond to climate change [57]. This research demonstrates high-throughput and highly sensitive measurements of the nutritional value of individual diatom cells, highlighting the potential for studies of macromolecular compositional changes in mixed natural populations, at the level of individual species [58]. Additionally, the single-cell approach has the ability to classify cells by taxon with sensitivity and specificity of up to 97.6% and 100%, respectively (see Table S2), corroborating studies with other prokaryotic and eukaryotic organisms [19,59]. Given that leading semi-automated plankton classification systems currently achieve accuracies of between 70-80%, incorporating the synchrotron FTIR approach into the taxonomic classification tool-box will facilitate progress in this field [60]. Moreover, increasing the efficiency of data collection by simultaneously measuring macromolecular composition and taxonomically classifying cells is particularly innovative and valuable.

This study identified species-specific variations in cellular levels of all four major classes of macromolecules (lipids, proteins, carbohydrates and phosphorylated molecules) in response to salinity and temperature regimes characteristic of sea ice, meltwater and pelagic habitats. For *F. cylindrus* and *C. simplex*, which belong to two of the most abundant genera in the Southern Ocean, lipid content was highest in cells from the sea ice treatment. Lipid accumulation has previously been reported in Antarctic sea ice algal communities due to the enhanced production of polyunsaturated fatty acids, resulting in a different quality of food being released into the food web in winter compared to other seasons [39]. Given that lipids have the highest caloric value of the macromolecular pools, the enhanced production of lipids by microalgae in sea ice habitats may also influence the quantity of calories available to the food web. Such changes in microalgal nutritional value may help to explain observations that summer krill densities within the southwest Atlantic Ocean correlate positively with sea-ice extent the previous winter [61]. However, since cellular protein content in *F. cylindrus* and *C. simplex* was lower in treatment conditions characteristic of the sea ice habitat, years with reduced duration and extent of sea ice may be associated with increased protein production by sea ice microalgae. Given that microalgae are the major primary source of protein for the Southern Ocean food web, a macromolecule that is essential for growth at higher trophic levels [62], the relationship between sea ice duration and extent, and microalgal protein production merits further investigation. Presently, the likelihood and potential consequences of such changes in macromolecular composition and energy production on the Southern Ocean ecosystem remain unknown.

## Supporting Information

Table S1 **Infrared Band Assignments.**
(DOCX)

Table S2 **PLSDA Classification by Species Summary Statistics.**
(DOCX)

## References

[1] Sarthou G, Timmermans KR, Blain S, Treguer P, Tréguer P (2005) Growth physiology and fate of diatoms in the ocean: a review. Journal of Sea Research 53: 25–42. doi:10.1016/j.seares.2004.01.007.

[2] Tang KW, Smith WO, Shields AR, Elliott DT (2009) Survival and recovery of Phaeocystis antarctica (Prymnesiophyceae) from prolonged darkness and freezing. Proc Biol Sci 276: 81–90. doi:10.1098/rspb.2008.0598. PubMed: 18765338. 18765338 PMC2614241

[3] Mock T, Kroon BMA (2002) Photosynthetic energy conversion under extreme conditions - I: important role of lipids as structural modulators and energy sink under N-limited growth in Antarctic sea ice diatoms. Phytochemistry 61: 41–51. doi:10.1016/S0031-9422(02)00216-9. PubMed: 12165301. 12165301

[4] Krell A, Beszteri B, Dieckmann G, Glockner G, Valentin K et al. (2008) A new class of ice-binding proteins discovered in a salt-stress-induced cDNA library of the psychrophilic diatom Fragilariopsis cylindrus (Bacillariophyceae). European Journal of Phycology 43: 423–433. doi:10.1080/09670260802348615.

[5] Diekmann ABS, Peck MA, Holste L, St John MA, Campbell RW (2009) Variation in diatom biochemical composition during a simulated bloom and its effect on copepod production. Journal of Plankton Research 31: 1391–1405. doi:10.1093/plankt/fbp073.

[6] Lizotte MP (2001) The contributions of sea ice algae to Antarctic marine primary production. American Zoologist 41: 57–73. Available online at: doi:10.1668/0003-1569(2001)041[0057:TCOSIA]2.0.CO;2

[7] Horner R, Ackley SF, Dieckmann GS, Gulliksen B, Hoshiai T et al. (1992) Ecology of sea ice biota 1. Habitat, Terminology and Methodology. Polar Biology 12: 417–427.

[8] Petrou K, Ralph PJ (2011) Photosynthesis and net primary productivity in three Antarctic diatoms: possible significance for their distribution in the Antarctic marine ecosystem. MEPS 437: 27–40. doi:10.3354/meps09291.

[9] Ralph PJ, Ryan KG, Martin A, Fenton G (2007) Melting out of sea ice causes greater photosynthetic stress in algae than freezing in. Journal of Phycology 43: 948–956. doi:10.1111/j.1529-8817.2007.00382.x.

[10] Agrawal AA (2001) Phenotypic Plasticity in the Interactions and Evolution of Species. Science 294: 321–326. doi:10.1126/science.1060701. PubMed: 11598291. 11598291

[11] Petrou K, Doblin M, Ralph P (2011) Heterogeneity in the photoprotective capacity of three Antarctic diatoms during short-term changes in salinity and temperature. Marine Biology 158: 1029–1041. doi:10.1007/s00227-011-1628-4.

[12] Fahl K, Kattner G (1993) Lipid content and fatty acid composition of algal communities in sea-ice and water from the Weddell Sea (Antarctica). Polar Biology 13: 405–409. doi:10.1007/BF01681982.

[13] Lee SH, Whitledge TE, Kang S-H (2008) Spring time production of bottom ice algae in the landfast sea ice zone at Barrow, Alaska. Journal of Experimental Marine Biology and Ecology 367: 204–212. doi:10.1016/j.jembe.2008.09.018.

[14] Whyte JNC (1987) Biochemical composition and energy content of six species of phytoplankton used in mariculture of bivalves. Aquaculture 60: 231–241. doi:10.1016/0044-8486(87)90290-0.

[15] Jakob T, Wagner H, Stehfest K, Wilhelm C (2007) A complete energy balance from photons to new biomass reveals a light- and nutrient-dependent variability in the metabolic costs of carbon assimilation. J Exp Bot 58: 2101–2112. doi:10.1093/jxb/erm084. PubMed: 17483116. 17483116

[16] Suárez I, Marañón E (2003) Photosynthate allocation in a temperate sea over an annual cycle: the relationship between protein synthesis and phytoplankton physiological state. Journal of Sea Research 50: 285–299. doi:10.1016/j.seares.2003.04.002.

[17] Murdock JN, Wetzel DL (2009) FT-IR Microspectroscopy Enhances Biological and Ecological Analysis of Algae. Applied Spectroscopy Reviews 44: 335–361. doi:10.1080/05704920902907440.

[18] Heraud P, Wood BR, Beardall J, McNaughton D (2007) Probing the influence of the environment on microalgae using infrared and raman spectroscopy. In: Kneipp K Aroca R Kneipp H Wentrup-Byrne E New Approaches in Biomedical Spectroscopy. American Chemical Society, Washington, DC; ETATS-UNIS (1974) (Revue), Vol. 963 pp. 85–106.

[19] Xie Y, Xu S, Hu Y, Chen W, He Y et al. (2012) Rapid identification and classification of staphylococcus aureus by attenuated total reflectance fourier transform infrared spectroscopy. Journal of Food Safety 32: 176–183. doi:10.1111/j.1745-4565.2012.00365.x.

[20] Heraud P, Wood BR, Tobin MJ, Beardall J, McNaughton D (2005) Mapping of nutrient-induced biochemical changes in living algal cells using synchrotron infrared microspectroscopy. FEMS Microbiol Lett 249: 219–225. doi:10.1016/j.femsle.2005.06.021. PubMed: 16006070. 16006070

[21] Twining BS, Baines SB, Fisher NS (2004) Element stoichiometries of individual plankton cells collected during the Southern Ocean Iron Experiment (SOFeX). Limnology and Oceanography 49: 2115–2128. doi:10.4319/lo.2004.49.6.2115.

[22] Heraud P, Stojkovic S, Beardall J, McNaughton D, Wood BR (2008) Intercolonial variability in macromolecular composition in P-starved and P-replete Scenedesmus populations revealed by infrared microscopy. Journal of Phycology 44: 1335–1339. doi:10.1111/j.1529-8817.2008.00564.x. 27041730

[23] Mock T, Junge K, Seckbach J (2007) Psychrophilic Diatoms. In: Seckbach J Algae and Cyanobacteria in Extreme Environments, Vol. 11 Springer Netherlands pp. 343–364. Available: 10.1007/978-1-4020-6112-7_18

[24] Armand LK, Crosta X, Romero O, Pichon J-J (2005) The biogeography of major diatom taxa in Southern Ocean sediments: 1. Sea ice related species. Palaeogeography, Palaeoclimatology, Palaeoecology 223: 93–126. doi:10.1016/j.palaeo.2005.02.015.

[25] Almandoz GO, Ferreyra GA, Schloss IR, Dogliotti AI, Rupolo V et al. (2008) Distribution and ecology of Pseudo-nitzschia species (Bacillariophyceae) in surface waters of the Weddell Sea (Antarctica). Polar Biology 31: 429–442. doi:10.1007/s00300-007-0369-9.

[26] Wood AM, Everroad R, Wingard L (2005) Measuring growth rates in microalgal cultures. In: Andersen RA Algal culturing techniques. Elsevier Academic Press pp. 269–286.

[27] Mock T, Valentin K (2004) Photosynthesis and Cold Acclimation: Molecular Evidence From a Polar Diatom. Journal of Phycology 40: 732–741. doi:10.1111/j.1529-8817.2004.03224.x.

[28] Wold S, Sjöström M, Eriksson L (2001) PLS-regression: a basic tool of chemometrics. Chemometrics and Intelligent Laboratory Systems 58: 109–130. doi:10.1016/s0169-7439(01)00155-1.

[29] Barker M, Rayens W (2003) Partial least squares for discrimination. Journal of Chemometrics 17: 166–173. doi:10.1002/cem.785.

[30] Bylesjö M, Rantalainen M, Cloarec O, Nicholson JK, Holmes E et al. (2006) OPLS discriminant analysis: combining the strengths of PLSDA and SIMCA classification. Journal of Chemometrics 20: 341. doi:10.1002/cem.1006.

[31] Jungandreas A, Wagner H, Wilhelm C (2012) Simultaneous measurement of the silicon content and physiological parameters by FTIR spectroscopy in diatoms with siliceous cell walls. Plant Cell Physiol 53: 2153–2162. doi:10.1093/pcp/pcs144. PubMed: 23104763. 23104763

[32] Wagner H, Liu Z, Langner U, Stehfest K, Wilhelm C (2010) The use of FTIR spectroscopy to assess quantitative changes in the biochemical composition of microalgae. J Biophotonics 3: 557–566. doi:10.1002/jbio.201000019. PubMed: 20503222. 20503222

[33] Giordano M, Kansiz M, Heraud P, Beardall J, Wood B et al. (2001) Fourier transform infrared spectroscopy as a novel tool to investigate changes in intracellular macromolecular pools in the marine microalga Chaetoceros muellerii (Bacillariophyceae). Journal of Phycology 37: 271–279. doi:10.1046/j.1529-8817.2001.037002271.x.

[34] Saibo NJM, Lourenço T, Oliveira MM (2009) Transcription factors and regulation of photosynthetic and related metabolism under environmental stresses. Ann Bot 103: 609–623. doi:10.1093/aob/mcn227. PubMed: 19010801. 19010801 PMC2707349

[35] Laws EA (1991) Photosynthetic quotients, new production and net community production in the open ocean. Deep Sea Research Part A Oceanographic Research Papers 38: 143–167. doi:10.1016/0198-0149(91)90059-O.

[36] Mock T, Kroon BMA (2002) Photosynthetic energy conversion under extreme conditions - II: the significance of lipids under light limited growth in Antarctic sea ice diatoms. Phytochemistry 61: 53–60. doi:10.1016/S0031-9422(02)00215-7. PubMed: 12165302. 12165302

[37] Falk-Petersen S, Sargent JR, Henderson J, Hegseth EN, Hop H et al. (1998) Lipids and fatty acids in ice algae and phytoplankton from the Marginal Ice Zone in the Barents Sea. Polar Biology 20: 41–47. doi:10.1007/s003000050274.

[38] Nichols PD, Palmisano AC, Rayner MS, Smith GA, White DC (1989) Changes in the lipid composition of Antarctic sea-ice diatom communities during a spring bloom: an indication of community physiological status. Antarctic Science 1: 133–140 Available: 10.1017/S0954102089000209

[39] Thomas DN, Dieckmann GS (2002) Antarctic sea ice-a habitat for extremophiles. Science 295: 641–644. doi:10.1126/science.1063391. PubMed: 11809961. 11809961

[40] Gwak I, sic Jung W, Kim H, Kang S-H, Jin E (2010) Antifreeze Protein in Antarctic Marine Diatom, Chaetoceros neogracile. Marine Biotechnology 12: 630–639. doi:10.1007/s10126-009-9250-x. 20024694

[41] Bayer-Giraldi M, Uhlig C, John U, Mock T, Valentin K (2010) Antifreeze proteins in polar sea ice diatoms: diversity and gene expression in the genus Fragilariopsis. Environ Microbiol 12: 1041–1052. doi:10.1111/j.1462-2920.2009.02149.x. PubMed: 20105220. 20105220

[42] Raymond JA (2000) Distribution and partial characterization of ice-active molecules associated with sea-ice diatoms. Polar Biology 23: 721–729. doi:10.1007/s003000000147.

[43] Devos N, Ingouff M, Loppes R, Matagne RF (1998) RUBISCO adaptation to low temperatures: a comparative study in psychrophilic and mesophilic unicellular algae. Journal of Phycology 34: 655–660. doi:10.1046/j.1529-8817.1998.340655.x.

[44] Laskowski RA (2001) PDBsum: summaries and analyses of PDB structures. Nucleic Acids Res 29 : 221–222. doi:10.1093/nar/29.1.221. PubMed: 11125097. 11125097 PMC29784

[45] Trovato M, Mattioli R, Costantino P (2008) Multiple roles of proline in plant stress tolerance and development. Rendiconti Lincei 19: 325–346. doi:10.1007/s12210-008-0022-8.

[46] Pocock T, Vetterli A, Falk S (2011) Evidence for phenotypic plasticity in the Antarctic extremophile Chlamydomonas raudensis Ettl. UWO 241. J Exp Bot 62: 1169–1177. doi:10.1093/jxb/erq347. PubMed: 21041369. 21041369 PMC3022403

[47] Green JL, Bohannan BJ, Whitaker RJ (2008) Microbial biogeography: from taxonomy to traits. Science 320: 1039–1043. doi:10.1126/science.1153475. PubMed: 18497288. 18497288

[48] Charmantier A, McCleery RH, Cole LR, Perrins C, Kruuk LE et al. (2008) Adaptive phenotypic plasticity in response to climate change in a wild bird population. Science 320: 800–803. doi:10.1126/science.1157174. PubMed: 18467590. 18467590

[49] Smetacek V, Nicol S (2005) Polar ocean ecosystems in a changing world. Nature 437: 362–368. doi:10.1038/nature04161. PubMed: 16163347. 16163347

[50] Arrigo KR (2005) Marine microorganisms and global nutrient cycles. Nature 437: 349–355. doi:10.1038/nature04159. PubMed: 16163345. 16163345

[51] Flynn KJ, Blackford JC, Baird ME, Raven JA, Clark DR et al. (2012) Changes in pH at the exterior surface of plankton with ocean acidification. Nature Climate Change 2: 510–513. doi:10.1038/nclimate1489.

[52] Arrigo KR (2012) Massive Phytoplankton Blooms Under Arctic Sea Ice. Science 336: 1408. doi:10.1126/science.1215065. PubMed: 22678359. 22678359

[53] Ducklow HW, Baker K, Martinson DG, Quetin LB, Ross RM et al. (2007) Marine pelagic ecosystems: the West Antarctic Peninsula. Philos Trans R Soc Lond B Biol Sci 362: 67–94. doi:10.1098/rstb.2006.1955. PubMed: 17405208. 17405208 PMC1764834

[54] Rivkin RB (1985) Carbon-14 labelling patterns of individual marine phytoplankton from natural populations. Marine Biology 89: 135–142. doi:10.1007/bf00392884.

[55] Rivkin RB, Voytek MA (1987) Photoadaptations of photosynthesis and carbon metabolism by phytoplankton from McMurdo Sound, Antarctica. 1. Species-specific and community responses to reduced irradiances. Limnology and Oceanography 32: 249–259. doi:10.4319/lo.1987.32.1.0249.

[56] Carr M-E, Friedrichs MAM, Schmeltz M, Noguchi Aita M, Antoine D et al. (2006) A comparison of global estimates of marine primary production from ocean color. Deep Sea Research Part II, Topical Studies in Oceanography 53: 741–770. doi:10.1016/j.dsr2.2006.01.028.

[57] Boyd PW, Strzepek R, Fu F, Hutchins DA (2010) Environmental control of open-ocean phytoplankton groups: Now and in the future. Limnology and Oceanography 55: 1353–1376. doi:10.4319/lo.2010.55.3.1353.

[58] Domenighini A, Giordano M (2009) Fourier Transform Infrared Spectroscopy of microalgae as a novel tool for biodiversity studies, species identification, and the asssessment of water quality. Journal of Phycology 45: 522–531. doi:10.1111/j.1529-8817.2009.00662.x. 27033830

[59] Naumann D, Helm D, Labischinski H (1991) Microbiological characterisations by FT-IR spectroscopy. Nature 351: 81–82. doi:10.1038/351081a0. PubMed: 1902911. 1902911

[60] MacLeod N, Benfield M, Culverhouse P (2010) Time to automate identification. Nature 467: 154–155. doi:10.1038/467154a. PubMed: 20829777. 20829777

[61] Atkinson A, Siegel V, Pakhomov E, Rothery P (2004) Long-term decline in krill stock and increase in salps within the Southern Ocean. Nature 432: 100–103. doi:10.1038/nature02996. PubMed: 15525989. 15525989

[62] Lohrenz SE, Taylor CD (1987) Primary production of protein: I. Comparison of net cellular carbon and protein synthesis with 14c-derived rate estimates in steady-state cultures of marine phytoplankton. Mar Ecol Prog Ser 35: 277–292. doi:10.3354/meps035277.

