# Supplementary material for: Phenotypic Plasticity of Southern Ocean Diatoms: Key to Success in the Sea Ice Habitat?
Source: PLoS One. 2013 Nov 21;8(11):e81185. doi: 10.1371/journal.pone.0081185 (PMC3868450; doi:10.1371/journal.pone.0081185)
Supplement: Table S1 — Infrared Band Assignments. (DOCX) [file pone.0081185.s001.docx]

Table S1 Infrared Band Assignments^[1](#_ENREF_1)^

| **Macromolecule** | **Wavenumber (cm-1)** | **Functional group** | **Comments** |
| --- | --- | --- | --- |
| Lipids | 3030 | C-H | From methine groups from unsaturated lipids |
| Lipids and proteins | 2960 | C-H | From methyl groups, generally dominated by proteins |
|  | 2920 | C-H | From methylene groups |
|  | 2855 | C-H | From methylene groups |
| Lipids | 1730 | C=O | From ester carbonyl groups of lipids |
| Proteins | 1655 | C=O | Amide I band from proteins |
|  | 1540 | C-H, N-H | Amide II band from proteins |
| Free amino acids | 1515 | C-C | From aromatic amino acids |
| Proteins and lipids | 1450 | CH_2_, CH_3_ | Bending vibrations mainly from proteins and lipids |
| Carboxylic acids | 1400 | COO^-^ | From carboxylate groups, mainly from amino acids |
| Phosphorylated molecules | 1240 | P=O | From the phosphodiester stretching vibrations of nucleic acids and other phosphorylated molecules |
| Carbohydrates | 1155 | C-O | Mainly from pyranose groups from carbohydrates |
| Silicate and | 1080 | Si-O | From the silicate frustule |
| phosphorylated molecules |  | P=O | Phosphorylated molecules |
| Carbohydrates | 1040 | C-O | From starch and sugar groups |

1 Heraud, P., Stojkovic, S., Beardall, J., McNaughton, D. & Wood, B. R. Intercolonial variability in macromolecular composition in P-starved and P-replete Scenedesmus populations revealed by infrared microscopy. *J. Phycol.* **44**, 1335-1339 (2008).
