# Supplementary material for: Phenotypic Plasticity of Southern Ocean Diatoms: Key to Success in the Sea Ice Habitat?
Source: PLoS One. 2013 Nov 21;8(11):e81185. doi: 10.1371/journal.pone.0081185 (PMC3868450; doi:10.1371/journal.pone.0081185)
Supplement: Table S2 — PLSDA Classification by Species Summary Statistics. (DOCX) [file pone.0081185.s002.docx]

Table S2 PLS-DA Classification by Species Summary Statistics

| Treatment | n | Species | R^2^ | Sensitivity | Specificity |
| --- | --- | --- | --- | --- | --- |
| Meltwater | 268 | *C. simplex* | 0.763 | 98.8% | 99.7% |
|  |  | *F. cylindrus* | 0.582 | 87.2% | 100.0% |
|  |  | *P. subcurvata* | 0.636 | 80.5% | 100.0% |
| Pelagic | 278 | *C. simplex* | 0.790 | 100.0% | 99.8% |
|  |  | *F. cylindrus* | 0.577 | 89.5% | 99.3% |
|  |  | *P. subcurvata* | 0.642 | 74.6% | 100.0% |
| Sea ice | 246 | *C. simplex* | 0.880 | 95.7% | 99.9% |
|  |  | *F. cylindrus* | 0.360 | 88.9% | 99.7% |
|  |  | *P. subcurvata* | 0.794 | 98.0% | 97.8% |
